# Variability in Fishmeal Nutritional Value in Weaned Pigs and Development of Predictive Equations

**DOI:** 10.3390/ani15131872

**Published:** 2025-06-24

**Authors:** Pei Yang, Xiaoyan Su, Bin Li, Junqi Jin, Bing Yu, Jun He, Jie Yu, Quyuan Wang, Huifen Wang, Daiwen Chen, Hui Yan

**Affiliations:** 1 Key Laboratory for Animal Disease-Resistance Nutrition of Ministry of Education, Institute of Animal Nutrition, Sichuan Agricultural University, Chengdu 611130, China; yangpei2669@163.com (P.Y.); su3414081@163.com (X.S.); 2023114024@stu.sicau.edu.cn (J.J.); ybingtian@163.com (B.Y.); hejun8067@163.com (J.H.); yujie@sicau.edu.cn (J.Y.); wangqy@sicau.edu.cn (Q.W.); wanghuifen1005@163.com (H.W.); 2 Sichuan Tequ Agriculture and Animal Husbandry Technology Group Co., Ltd., Chengdu 610207, China; binli2020@foxmail.com

**Keywords:** fishmeal, piglets, ileal amino acid digestibility, effective energy, effective nutrient prediction

## Abstract

The apparent ileal digestibility (AID) and standardized ileal digestibility (SID) of amino acids, digestible energy (DE), metabolizable energy (ME), and the apparent total tract digestibility (ATTD) of nutrients in 10 fishmeal (FM) samples were evaluated in weaned barrows (Duroc × Landrace × Yorkshire) using two experiments. In Experiment 1, 11 piglets (18.87 ± 0.10 kg) fitted with T-cannulas were randomly allocated to an 11 × 6 Latin-square design with 11 diets (1 nitrogen-free diet and 10 assay diets) and six periods. The AID and SID of all amino acids (AAs) except proline showed significant differences among all FM (*p* < 0.05). Importantly, the SID of amino acids was positively correlated with key antioxidant markers and immune parameters, and it was negatively correlated with oxidative stress markers (MDA) and pro-inflammatory cytokines (IL-2 and IL-6). In Experiment 2, 11 piglets (18.05 ± 1.15 kg) were assigned to an 11 × 5 Latin-square design with 11 diets (a 96.35% corn diet and 10 assay diets) and five consecutive periods. Significant variations were observed in the DE, ME, and ATTD of dry matter among different FM samples (*p* < 0.05). Moreover, predictive equations for estimating the SID of lysine, methionine, threonine, and tryptophan, as well as DE and ME, were established using stepwise regression analysis based on the chemical composition of the FM. These findings demonstrate that the nutritional value of FM in nursery pig diets has been underestimated, and this study provides precise data and predictive methods for evaluating the nutritional quality of FM in precision nutrition.

## Simple Summary

Fishmeal (FM) is a valuable ingredient in piglet diets, offering high-quality protein and essential nutrients that promote growth and intestinal health. However, current nutritional data for FM, particularly for weaned piglets, is limited, and there are no accurate models to predict its nutrient utilization in real time. This study evaluates the digestibility and energy values of 10 different FM varieties in piglets, revealing significant variations in nutrient absorption. The results also show that the current NRC estimates for FM’s nutritional value may be underestimated. Additionally, the study develops predictive models to estimate the digestibility of key amino acids and energy values based on FM’s chemical composition. These findings provide crucial data and predictive tools to improve the precision of FM usage in animal feeding, advancing the development of more efficient and tailored diets for weaned pigs.

## 1. Introduction

Fishmeal (FM) is widely recognized as a high-quality animal feed, particularly for animals in early growth stages, owing to its rich protein content and well-balanced profile of amino acids (AAs), vitamins, and minerals [1,2]. FM is widely used in creep and nursery diets, especially during the early post-weaning period, primarily to stimulate feed intake, promote growth, and enhance immunity. In creep feed, the recommended inclusion level of fishmeal is typically from 3% to 6%. In swine production, FM is especially beneficial for piglets and early-growing pigs, whose gastrointestinal systems are still developing and are less tolerant of anti-nutritional factors commonly found in plant-based protein sources such as soybean meal and rapeseed meal, including trypsin inhibitors [3]. Studies have demonstrated that FM promotes villus development in the small intestine, potentially enhancing intestinal health and improving nutrient digestibility [4,5]. However, the nutritional value and quality of FM can vary significantly due to differences in the fish species used and the production processes involved, such as deoiling, dehydrating, and crushing [2,6]. Furthermore, while the Nutrient Requirements of Swine (NRC, 2012) [7] provides nutrient values primarily based on growing pigs, FM is more prevalently used in piglet diets—yet there remains a lack of precise digestibility data and energy values for this age group. To our knowledge, limited studies have systematically evaluated the digestibility and energy utilization of multiple FM sources specifically in weaned pigs.

In this context, our study is novel in that it not only evaluates the standardized ileal digestibility (SID) of crude protein (CP) and amino acids (AAs), as well as the digestible energy (DE), metabolizable energy (ME), and apparent total tract digestibility (ATTD) of nutrients from 10 different FM sources in weaned pigs—but also derives specific prediction equations for SID of limiting amino acids (LAAs), DE, and ME. These models aim to support accurate dietary formulation and enhance the application of FM in precision piglet nutrition.

## 2. Materials and Methods

The Institutional Animal Care and Use Committee of Sichuan Agricultural University (No.20200058) approved the protocols for animal trials conducted with Duroc × Landrace × Yorkshire barrows. Identical batches of FM sourced from Peru (FM 1–6), the United States (FM 7), and China (FM 8–10) were utilized in both experiments (Table 1). The research facility maintained a controlled environment with a temperature of 26 °C and a relative humidity of 50%. Pigs were housed individually in metabolism cages (2.5 m × 1.8 m × 0.8 m) and had free access to water.

### 2.1. Experiment 1: Ileal Digestibility of CP and AAs

#### 2.1.1. Diets, Animals, and Experimental Design

Eleven barrows (initial body weight: 18.87 ± 0.10 kg) were surgically fitted with a simple T-cannula at the distal ileum following the procedures outlined by Stein [8]. A 14-day recovery period was implemented post-surgery to ensure animal welfare. The experiment utilized a set of 11 diets, evaluated in six congruent 11 × 6 Latin square designs, with 11 pigs assigned to each design. A nitrogen (N)-free diet was used to determine the basal endogenous losses of CP and AAs [9]. Ten experimental diets were formulated with FM as the sole N source, adjusted to approximately 16% CP (Table 2), based on the varying CP levels of the FM samples (Table 1). All diets contained 0.3% chromic oxide (Cr_2_O_3_) as an indigestible marker and were supplemented with vitamins and minerals to meet the nutrient requirements for nursery pigs [7].

#### 2.1.2. Experimental Procedure

Pigs were fed at a daily rate of 2.5 times the estimated maintenance energy requirement. Each experimental period consisted of a 3-day adaptation phase to the diets, a 3-day of ileal digesta collection, followed by 5-day of recovery phase with commercial diets. With a total of 6 experimental periods, the entire study lasted 66 days. Digesta samples were collected 2 h post-feeding using plastic bags attached to the cannula barrel, with the bags being replaced every 20–30 min [10]. During the ileal digesta collection period, approximately 800–1000 mL of digesta was collected per pig. At the end of each experimental period, approximately 40 mL of blood was collected from the anterior vena cava, centrifuged (3500× *g*, 4 °C, 15 min) to isolate the serum. The samples were stored at −20 °C for approximately two months before further analysis [11].

#### 2.1.3. Chemical Analysis

Pooled digesta samples from each animal and period were thawed, homogenized, and ground using an oscillating disk mill to pass through a 60-mesh sieve for analysis. Ingredient, diet, and ileal digesta samples were analyzed in duplicate for DM (Method 930.15), CP (Method 984.13), EE (Method 920.39), ash (Method 942.05), Ca (Method 978.02), and P (Method 946.06), following the procedures of AOAC International (2019) [12]. Diet and digesta samples were analyzed for 15 amino acids (AAs), excluding methionine (Met), cysteine (Cys), and tryptophan (Trp), via 24 h hydrolysis with 6 mol/L HCl at 110 °C. Trp was determined by high-performance liquid chromatography following hydrolysis with LiOH at 110 °C for 22 h. Met and Cys were determined using HPLC (Agilent 1200 Series, Agilent, Santa Clara, CA, USA) after oxidation with formic acid for 18 h and subsequent hydrolysis with 7.5 mol/L HCl at 110 °C for 24 h. CP was calculated as analyzed N × 6.25. Chromium (Cr) concentrations were determined using an atomic absorption spectrophotometer (Z-5000, Hitachi, Tokyo, Japan).

#### 2.1.4. Serum Antioxidation and Immune Analyses

The levels of antioxidant capacity (T-AOC), glutathione peroxidase (GPX-Px), total superoxide dismutase (T-SOD), total catalase (CAT), and malondialdehyde (MDA) were determined by ELISA kits (Nanjing Jiancheng Bioengineering Institute, Nanjing, China). The total serum concentrations of immunoglobulin (Ig) subsets (A, M, and G), interleukin 2 (IL-2), interleukin 6 (IL-6), and interferon-γ (IFN-γ) were measured by ELISA (Jiangsu Enzyme Immunity Industry Co., Ltd., Yancheng, China).

#### 2.1.5. Calculation and Statistical Analysis

Ileal endogenous losses, apparent ileal digestibility (AID), and the SID of CP and AAs were calculated [13]. Pearson correlation analysis was performed in SAS to assess the relationship between SID and antioxidant capacity and immune function, with results visualized using a heat map in GraphPad Prism 8.0.1. Data were analyzed using the GLM procedure in SAS (version 9.4, SAS Institute Inc., Cary, NC, USA). The statistical model used was the following: Yij = μ + αi + γj + εij, where μ is the overall mean, αi is the treatment effect, γj is the block effect, and εij is the error term. Results are presented as least squares means with pooled SEM. Statistical significance was set at *p* < 0.05, with trends considered at 0.05 ≤ *p* ≤ 0.10. Stepwise regression in the REG procedure was used to develop prediction equations for the SID of lysine (Lys), methionine (Met), threonine (Thr), and tryptophan (Trp), with chemical components of FM as predictor variables. Only variables with *p* < 0.05 were retained in the model. The fit of the equations was evaluated using R^2^ and *p*-values.

### 2.2. Experiment 2: Total Tract Digestibility and Energy Concentration

#### 2.2.1. Diets, Animals, and Experimental Design

Eleven barrows (18.05 ± 1.15 kg) were randomly assigned to an 11 × 5 Latin-square design, with 11 diets and 5 consecutive periods. One basal diet consisted of 96.35% corn, while 10 FM diets (Table 3) were formulated to provide equal protein levels based on the CP content of FM as specified in Table 1. Vitamins and minerals were supplemented to meet the nutritional requirements for nursery pigs [7].

#### 2.2.2. Experimental Procedure

The pigs were weighed prior to the initiation of each experimental period, and the feed allowance was equivalent to 4% of body weight and divided into 3 equal meals per day. Each period included 4 days of diet adaptation followed by 5 days of total feces and urine collection. With a total of 5 experimental periods, the entire study lasted 45 days. Ferric oxide and Cr_2_O_3_ were added into diets once to mark the start and end of fecal collection, respectively [14]. Feces were frozen at −20 °C immediately after collection. Urine was collected in buckets with 50 mL of 10% H_2_SO_4_ as a preservative, and 10% of urine sample were stored at −20 °C. A total of 500 g of fecal samples and 100 mL of urine samples were collected.

#### 2.2.3. Chemical Analyses

Fecal and urine samples were pooled for each collection period. Fecal samples were dried at 65 °C for 72 h, then ground to pass through a 40-mesh sieve for analysis. As in Experiment 1, fecal and diet samples were analyzed in duplicate for DM (Method 930.15), CP (Method 984.13), EE (Method 920.39), ash (Method 942.05), Ca (Method 978.02), and P (Method 946.06), while urine samples were analyzed in duplicate for Kjeldahl N. Gross energy (GE) was measured in all samples using a bomb calorimeter (Parr Instrument Co., Moline, IL, USA)

#### 2.2.4. Calculation and Statistical Analysis

The DE, ME, and ATTD of DM, CP, EE, ash, Ca, and P for FM were calculated according to reported method [14].

$$DE_{d}=\frac{GE_{i}-CE_{f}}{F_{i}}$$

$$DE_{i}=\frac{DE_{d}\;in\;FM\;energy\;diet-A\times DE_{d\;}in\;basal\;diet}{B}$$

$$ME_{d}=\frac{GE_{i}-CE_{f}-CE_{u}}{F_{i}}$$

$$ME_{i}=\frac{ME_{d}\;in\;FM\;energy\;diet-A\times ME_{d\;}in\;basal\;diet}{B}$$

$$ATTD\;nutrients\;in\;FM=100\times \frac{{T\times T}_{P}-{B\times B}_{P}}{{FM}_{P}}$$

where *DE_d_* and *Me_d_* are the DE and ME values in each experimental energy diet, *De_i_* and *Me_i_* are the DE and ME values in FM, *GE_i_* is calculated as the product of the actual feed intake and the GE content of each experimental energy diet over the 5d collection period, and *GE_f_* and *GE_u_* are the GE content in feces and urine of each pig over the 5d collection period.

The ATTD of nutrients in FM represents the digestibility (%) of the nutrient in FM, where *T* is the overall digestibility of the nutrient in the test diet, *B* is the digestibility of the nutrient in the basal diet, and *Tₚ* is the total proportion of the nutrient in the test diet (*Tₚ = Bₚ + FMₚ* = 100%). *Bₚ* is the proportion (%) of the nutrient in the test diet that is contributed by the basal diet, and *FMₚ* is the proportion (%) contributed by FM.

Data were analyzed using the GLM procedure in SAS (version 9.4, SAS Institute Inc., Cary, NC, USA). The statistical model used was the following: *Yij = μ + αi + γj + εij*, where μ is the overall mean, *αi* is the treatment effect, *γj* is the block effect, and *εij* is the error term. Results are presented as least squares means with pooled SEM. Statistical significance was set at *p* < 0.05, with trends considered at 0.05 ≤ *p* ≤ 0.10. Stepwise regression using the REG procedure was employed to develop prediction equations for DE and ME, with the chemical components of FM as predictor variables. Only variables with *p* < 0.05 were retained in the final model. The goodness of fit was evaluated using R^2^ values and the corresponding *p*-values.

## 3. Results

### 3.1. Experiment 1: Ileal Digestibility of CP and AAs

#### 3.1.1. Chemical Composition in FM

The chemical composition profiles of the 10 FM samples were analyzed, revealing substantial consistency with the values reported by NRC (2012) [7] (Table 4). The mean DM content across the 10 FM variants was 92.4%, ranging from 90.90% to 93.70%, with a coefficient of variation (CV) of less than 1%. The mean CP content was 64.19%, exhibiting a relatively low CV of 5.60%. The average levels of EE, ash, Ca, and P were 10.27%, 18.45%, 4.01%, and 2.73%, respectively, with CVs exceeding 10% ranging from 16.14% to 26.62%. The LAAs, including Lys, Met, Thr, and Trp, averaged 4.71%, 1.72%, 2.63%, and 0.60%, respectively. The CVs for histidine (His), Trp, cysteine (Cys), glycine (Gly), and proline (Pro) exceeded 10%, ranging from 10.84% to 25.62% (Table 4).

#### 3.1.2. Apparent Ileal Digestibility and Standardized Ileal Digestibility of CP and AAs in FM

Significant variations in the AID and SID of CP and AAs were observed among the various FM samples (*p* < 0.05), with the exception of Pro, which showed no significant differences (*p* > 0.05). Comparative analysis of CP and AA digestibility across FM samples indicated that FM 8 exhibited the lowest ileal digestibility while FM 6 demonstrated the highest (*p* < 0.05) (Table 5 and Table 6). The mean AID values for CP and Pro in FM were notably lower than the NRC reference values, particularly for Pro, which displayed a negative range (*−*29.38% to 29.90%) and a high standard error of the mean (SEM) of 34.34% (Table 5). Although the mean AID values for most AAs were generally consistent with the NRC values, some were found to be lower (Table 5). In contrast, the mean SID values for CP and AAs in FM exceeded the NRC values (Table 6). Specifically, the AID values for CP, Lys, Met, Thr, and Trp across the 10 FM samples were 68.49% (range: 48.01% to 77.86%), 85.77% (range: 76.53% to 90.88%), 86.91% (range: 77.70% to 92.10%), 76.52% (range: 60.93% to 85.73%), and 71.33% (range: 53.04% to 80.35%), respectively (Table 5). Similarly, the SID values for these components were 86.99% (range: 66.26% to 97.47%), 91.20% (range: 82.03% to 96.33%), 91.25% (range: 82.64% to 95.58%), 89.24% (range: 73.55% to 98.35%), and 84.34% (range: 65.23% to 94.41%), respectively (Table 6).

#### 3.1.3. Prediction Equations for SIDLAA

Stepwise regression equations were developed to predict the SIDLAA in FM. Among the representative chemical constituents, arginine (Arg) exhibited a positive correlation with Lys, Met, Thr, and Trp (*p* < 0.05). Cys was identified as the best predictor for SIDLys through linear stepwise regression analysis (Table 7). The best-fit equations for SID were as follows: SIDLys (%) = 33.93 + 11.54 × Arg (%) + 26.41 × Cys (%) (R*^2^* = 0.91, *p* < 0.05); SIDMet (%) = 54.21 + 10.51 × Arg (%) (R*^2^* = 0.57, *p* < 0.05); SIDThr (%) = 25.11 + 18.01 × Arg (%) (R*^2^* = 0.53, *p* < 0.05); and SIDTrp (%) = 6.15 + 22.08 × Arg (%) (R*^2^* = 0.54, *p* < 0.05). Additionally, a recommended equation for SIDLys was proposed as follows: SIDLys (%) = 29.00 + 8.31 × Arg (%) + 37.87 × Cys (%) + 3.35 × Pro (%) (R*^2^* = 0.96, *p* < 0.05) (Table 7). These equations demonstrate strong predictive accuracy and highlight the significant influence of Arg and Cys on the digestibility of key amino acids in FM.

#### 3.1.4. Antioxidant and Immune Capacity in Serum

**Figure 1:**
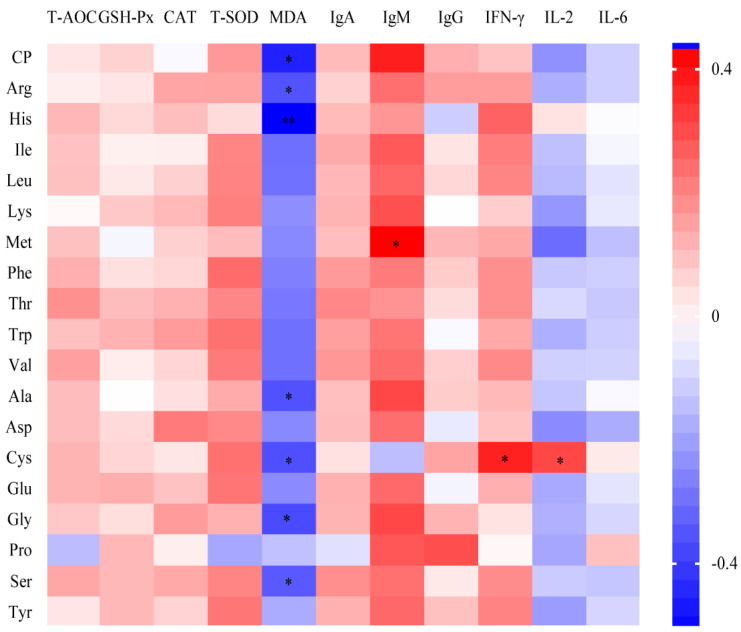
Spearman correlation analysis of SID values with antioxidant capacity and immune function in the experiment. In the heatmap, red indicates a positive correlation, while blue represents a negative correlation. Color intensity represents R-values of correlation, and the scale of the colors is denoted as follows: a positive correlation is represented by a darker shade of red, while a negative correlation is represented by a darker shade of blue. *, 0.01 ≤ *p* < 0.05; **, *p* < 0.01.

The effects of different FM sources on antioxidant and immune capacity in serum were evaluated. Pigs fed FM 8 exhibited a trend toward increased serum MDA concentrations (*p* = 0.05). FM 2 significantly elevated IgM levels (*p* < 0.05), while FM 5 increased IgG levels (*p* < 0.05) compared with FM 7 and FM 10 (Table 8). Additionally, FM 4, FM 6, and FM 9 significantly enhanced serum IFN-γ levels (*p* < 0.05). In contrast, FM 2, FM 3, and FM 7 led to a significant reduction in serum IL-2 levels (*p* < 0.05), whereas FM 2 and FM 7 also decreased serum IL-6 levels (*p* < 0.05) (Table 8). Spearman correlation analysis further demonstrated associations between SID values and serum antioxidant and immune function indicators. A positive correlation was observed between the SID of CP and AAs and the serum levels of T-AOC, GSH-Px, CAT, T-SOD, IgA, IgM, IgG, and IFN-γ, whereas a negative correlation was noted with MDA, IL-2, and IL-6. Specifically, the IgM level exhibited a significant positive correlation with SIDMet (*p* < 0.05), while IFN-γ was positively associated with SIDCys (*p* < 0.05). Moreover, MDA content showed a significant negative correlation with the SIDs of CP, Arg, His, Alanine (Ala), Cys, Gly, and Serine (Ser) (*p* < 0.05) (Figure 1).

### 3.2. Experiment 2: Total Tract Digestibility and Energy Concentration

#### 3.2.1. ATTD of Nutrients in FM

As shown in Table 9, significant differences were observed in the ATTD of DM, CP, and ash among various FM samples (*p* < 0.05), with FM 10 exhibiting the highest ATTD (*p* < 0.05) and FM 7 showing the lowest (*p* < 0.05). The mean ATTD values for DM, CP, EE, ash, Ca, and P were 78.96% (range: 71.09% to 84.54%), 92.69% (range: 89.58% to 94.17%), 74.67% (range: 69.31% to 83.58%), 62.41% (range: 46.58% to 74.03%), 52.09% (range: 40.34% to 62.41%), and 65.44% (range: 55.88% to 74.41%), respectively. Notably, the mean ATTD of P was 20.72% lower than the value recommended by the NRC.

#### 3.2.2. Energy and N Balance in Piglets Fed Different FM Diets

In the evaluation of N and energy balance in piglets fed different FM diets, no significant differences were observed in N intake, fecal N, urinary N, N deposition, N deposition rate, or biological value (BV) among these FM (*p* > 0.05) (Table 10). Similarly, daily fecal and urinary outputs, GE intake, and GE in feces and urine showed no significant variations among piglets consuming FM-based diets (*p* > 0.05) (Table 11).

#### 3.2.3. DE, ME, and ATTD of GE in FM

Significant variations were detected in the DE, ME, and ATTD of GE across different FM samples (*p* < 0.05) (Table 12). Specifically, FM 4 and FM 10 demonstrated the highest levels of DE, ME, and ATTD of GE (*p* < 0.05), while FM 9 exhibited the lowest DE and ME (*p* < 0.05), and FM 7 showed the lowest ATTD of GE (*p* < 0.05). The average values for DE, ME, ATTD of GE, and the ME/DE ratio were 3.69 Mcal/kg (range: 3.38 to 3.91 Mcal/kg), 3.58 Mcal/kg (range: 3.25 to 3.80 Mcal/kg), 82.83% (range: 76.21% to 89.60%), and 96.86% (range: 95.88% to 97.87%), respectively (Table 12). The DE in FM was lower than the NRC reference value, while the ME was slightly higher, resulting in a greater ME/DE ratio compared to the NRC recommendation.

#### 3.2.4. Prediction Equations for DE and ME of FM

Stepwise regression analysis improved the precision of prediction equations for DE and ME. The best-fit models included CP, EE, and P, with P showing a negative correlation with DE and ME (*p* < 0.05) (Table 13). The highest coefficients of determination were achieved for the following prediction models: DE (Mcal/kg) = 2.51 *−* 0.29 × P (%) + 0.03 × CP (%) (R*^2^* = 0.90, *p* < 0.05) and ME (Mcal/kg) = 1.75 *−* 0.39 × P (%) + 0.04 × CP (%) + 0.04 × EE (%) (R*^2^* = 0.95, *p* < 0.05). Cys emerged as the best predictor in multiple linear regression models incorporating amino acids; however, its inclusion reduced the accuracy of the prediction models as follows: DE (Mcal/kg) = 1.86 + 3.02 × Cys (%) (R*^2^* = 0.86, *p* < 0.05) and ME (Mcal/kg) = 1.66 + 3.17 × Cys (%) (R*^2^* = 0.84, *p* < 0.05).

## 4. Discussion

### 4.1. Ileal AA Digestibility of FM

The assessment of essential amino acid (EAA) requirements in piglets must account for their proportional relationship with LAAs, as this directly impacts growth performance and feed cost efficiency [15,16]. While the chemical composition of the FM in our study aligned with NRC (2012) standards [7], we observed a higher CV for EAA content. Notably, the AID of CP and select LAAs in FM was lower than the NRC (2012) [7] reference values but consistent with findings in weanling pigs by Rojas and Stein [17]. Conversely, the SID values for LAAs exceeded the NRC (2012) estimates, corroborating earlier reports [18]. This discrepancy suggests that current NRC SID values for FM may underestimate its true digestibility in piglet diets.

The observed variability in nutrient digestibility is primarily attributed to intestinal health in nursery pigs, which are more susceptible to a range of stressors that can lead to intestinal damage. Such damage results in increased endogenous N loss. Intestinal mucosal detachment, commonly seen in simple-stomached animals, has been shown to elevate endogenous protein excretion, leading to a reduction in AID [19]. This condition may explain why, in our study, the AID values for CP and AAs were lower than NRC (2012) [7] estimates, while the SID values were higher.

The implantation of a T-cannula in the distal ileum caused intestinal injury and oxidative stress in pigs [20,21], which provided the model to investigate the interplay between physiological stress and nutrient utilization. In response to such stressors, animals typically upregulate antioxidant enzymes (e.g., T-AOC, CAT, SOD, GSH-Px) and reduce MDA levels, a marker of lipid peroxidation [22,23,24]. Notably, our study detected positive correlations between activities of antioxidant enzymes and the SID of most AAs and inverse correlations between MDA levels and SID values, suggesting that the antioxidation capacity of piglets was critical for intestinal integrity and AA digestibility. Furthermore, immune modulation plays a pivotal role in shaping AA absorption efficiency. Immunoglobulins, such as IgM, enhance gut barrier function by neutralizing pathogens and reducing antigenic load [25]. The positive correlation between immunoglobulins, particularly IgM, and the SID of AAs in our study revealed the protective role of immune components in nutrient utilization. Baseline levels of IFN-γ are crucial for the initial recognition and clearance of viruses. Our study found a positive correlation between interferon levels and the SID of AAs, indicating that maintaining adequate interferon levels is important for controlling viral replication and supporting gut health and nutrient absorption [26,27]. Conversely, chronic inflammation has been reported to negatively correlate with intestinal function, a finding confirmed by our study, which showed that IL-2 levels were inversely correlated with the SID of AAs [28].

Stepwise regression identified Arg as the primary predictor for SID of LAAs (SIDLAA), underscoring its dual role in protein synthesis and metabolic regulation [29]. For SIDLys, SIDCys exhibited a higher regression coefficient than Arg, though its low FM content limits practical significance. The robust predictive equation for SIDLys (SIDLys (%) = 33.93 + 11.54 × Arg (%) + 26.41 × Cys (%), R^2^ = 0.91) highlights Arg’s dominance in enhancing lysine digestibility. However, lower R^2^ values for SIDMet, SIDThr, and SIDTry models (R^2^ < 0.85) emphasize the need for expanded datasets to improve accuracy.

### 4.2. The ATTD, DE, and ME of Different FM

The FM used in both experiments originated from the same batch. The evaluation of ATTD of energy in FM from Experiment 2 largely corresponded with the ileal AA digestibility findings from Experiment 1. However, the DE content of FM in the current study was slightly lower than published values [4,7,30], which aligns with the observations of Rojas and Stein [17], possibly due to the higher ash content in the FM used. In contrast, the mean ME in FM was slightly higher than the NRC (2012) [7] estimates and approached values reported by Kong [4], although it was lower than the figures provided by Kim for growing pigs [30]. Additionally, the ATTD of GE was lower than that reported by Rojas and Stein, and the ME/DE ratio was higher compared to the NRC (2012) [7] reference. These differences likely indicate higher urinary energy losses, which may be influenced by factors such as variations in the sources of fish or fishery by-products used to produce FM, as well as differences in manufacturing processes [31]. Furthermore, energy utilization from FM may also be affected by the growth stage of animals, with nursery pigs generally showing slightly lower energy digestibility compared to grow-finish pigs [32].

Our study highlighted the substantial variability in DE and ME across different FM samples. We developed predictive equations for DE and ME based on variations in nutrient content, allowing the fast identification of the quality of FM. We found that CP and P content emerged as the primary factors influencing the digestive and metabolic energy of FM. Specifically, higher CP levels and lower P levels contributed to greater available energy, consistent with the findings of Ouyang [33], who reported a decrease in DE and ME as P content increased. When recruiting AAs in stepwise regression, Cys content was a key factor influencing both DE and ME. Zong demonstrated that sulfur amino acids enhanced the activity of digestive enzymes in the jejunum, thereby improving nutrient absorption and utilization [34]. Our findings were consistent with this, showing a positive correlation between Cys content and both DE and ME in FM. The predictive equations for DE and ME, characterized by high R^2^ values (R^2^ = 0.86 and R^2^ = 0.84, respectively), effectively captured the variability in energy digestibility across different FM sources. It is worth noting that these predictive equations were developed based on FM samples commonly used in China. Due to potential differences in fish species and processing technologies across countries, the applicability of these equations to FM produced in other regions may be limited. This highlights the need for further validation before the equations can be widely applied in international contexts.

## 5. Conclusions

Our findings collectively reveal significant disparities in nutrient digestibility among FM sources, highlighting that the NRC (2012) [7] values likely underestimate the SID of amino acids and ME in FM for nursery pigs. The predictive equations for SIDAA, DE, and ME enabled the rapid assessment of FM quality, supporting informed decision making in feed formulation. The interplay between oxidative stress, immune status, and intestinal health directly governs amino acid digestibility, emphasizing the need for holistic health management to maximize nutrient utilization.

## Figures and Tables

**Table 1:** Sources of fishmeal (FM).

No.	CP, %	Source	Production Site	Processing	Specie
FM 1	68	Peru	PESQUERA EXALMAR S.A.A. (Lima, Peru)	Cooking	Peruvian anchovy
FM 2	68	Peru	TASA (Lima, Peru)	Cooking	Peruvian anchovy
FM 3	68	Peru	PESQUERA HAYDUK S.A. (Lima, Peru)	Steam drying	Peruvian anchovy
FM 4	68	Peru	Corporacion Pesquera Inca S.A.C.-Chancay Plant (Lima, Peru)	Cooking	Peruvian anchovy
FM 5	65	Peru	TASA (Lima, Peru)	Cooking	Peruvian anchovy
FM 6	65	Peru	Corporacion Pesquera Inca S.A.C.-Chancay Plant (Lima, Peru)	Cooking	Peruvian anchovy
FM 7	64	America	-	Cooking	American herring
FM 8	60	China	Tianjin Hengyuan Feed Sales Co., Ltd. (Tianjin, China)	Steam drying	Sea miscellaneous fish
FM 9	60	China	Cangzhou Qiankun Feed Sales Co., Ltd. (Cangzhou, China)	Degreasing	Sea miscellaneous fish
FM 10	60	China	Hebei Haixing Fish Meal Feed Factory (Hebei, China)	Degreasing	Sea miscellaneous fish

**Table 2:** Ingredient composition of diets in experiment 1 ^1^ (as-fed basis).

Items	DNF	FM			
		68%	65%	64%	60%
Ingredients, %					
Corn starch	79.20	58.60	58.10	57.10	55.60
Fishmeal	-	23.50	24.00	25.00	26.50
Sucrose	10.00	10.00	10.00	10.00	10.00
Soya-bean oil	3.00	3.00	3.00	3.00	3.00
CMC	4.00	4.00	4.00	4.00	4.00
Calcium carbonate	0.50	-	-	-	-
Dicalcium phosphata	1.90	-	-	-	-
Cr_2_O_3_	0.30	0.30	0.30	0.30	0.30
NaCl	0.40	0.40	0.40	0.40	0.40
Vitamin premix ^1^	0.05	0.05	0.05	0.05	0.05
Mineral premix ^2^	0.15	0.15	0.15	0.15	0.15
K_2_CO_3_	0.40	-	-	-	-
MgO	0.10	-	-	-	-
Total	100.00	100.00	100.00	100.00	100.00
Nutrient level ^3^					
DE, Mcal/Kg	3.81	3.74	3.74	3.73	3.72
ME, Mcal/Kg	3.66	3.49	3.49	3.48	3.46
CP, %	0.02	16.00	16.10	16.02	15.92
Ca, %	0.50	1.07	1.10	1.14	1.21
P, %	0.43	0.69	0.71	0.74	0.78
SID Lys, %	-	1.00	1.03	1.07	1.13
Digestible met, %	-	0.38	0.39	0.40	0.43
Digestible Thr, %	-	0.52	0.53	0.55	0.59
Digestible Trp, %	-	0.14	0.14	0.15	0.16

^1^ The vitamin premix provided the following per kg of the diet: VA, 15,000 IU; VB_1_, 5 mg; VB_2_, 12.5 mg; VB_6_, 6 mg; VB_12_, 0.06 mg; VD_3_, 5000 IU; VE, 40 IU; VK_3_, 5 mg; D-biotin, 0.25 mg; folic acid, 2.5 mg; D-pantothenic acid, 25 mg; and nicotinamide, 50 mg. ^2^ Mineral premix provided the following per kg of diets: Fe (FeSO_4_·H_2_O), 100.0 mg; Cu (CuSO_4_·5H_2_O), 6.0 mg; Zn (ZnSO_4_·H_2_O), 80.0 mg; Mn (MnSO_4_·H_2_O), 3.0 mg; I (KI), 0.15 mg; and Se (Na_2_SeO_3_), 0.26 mg. ^3^ Nutrient levels were the calculated values.

**Table 3:** Ingredient composition of diets in experiment 2 ^1^ (as-fed basis).

Ingredients	Corn Group	FM			
		68%	65%	64%	60%
Ingredients, %					
Corn	96.35	71.35	70.55	70.05	67.95
Fishmeal	-	25.00	25.80	26.30	28.40
Calcium carbonate	1.05	1.05	1.05	1.05	1.05
Dicalcium phosphate	1.40	1.40	1.40	1.40	1.40
NaCl	0.30	0.30	0.30	0.30	0.30
L-Lys·HCl	0.45	0.45	0.45	0.45	0.45
L-Thr	0.05	0.05	0.05	0.05	0.05
DL-Met	0.10	0.10	0.10	0.10	0.10
Vitamin premix ^1^	0.15	0.15	0.15	0.15	0.15
Mineral premix ^2^	0.15	0.15	0.15	0.15	0.15
Total	100.00	100.00	100.00	100.00	100.00
Nutrient level ^3^					
DE, Mcal/Kg	3.30	3.25	3.24	3.24	3.24
ME, Mcal/Kg	3.22	3.05	3.05	3.04	3.03
CP, %	7.71	22.46	22.41	22.44	22.48
Ca, %	0.72	1.86	1.89	1.92	2.01
TP, %	0.51	1.16	1.19	1.20	1.25
AP, %	0.30	1.01	1.03	1.04	1.10
Digestible Lys, %	0.44	1.55	1.58	1.60	1.69
Digestible Met, %	0.14	0.51	0.52	0.53	0.56
Digestible Thr, %	0.26	0.72	0.74	0.75	0.79

^1^ The vitamin premix provided the following per kg of the diet: VA, 15,000 IU; VD_3_, 5000 IU; VE, 40 IU; VK_3_, 5 mg; VB_1_, 5 mg; VB_2_, 12.5 mg; VB_6_, 6 mg; VB_12_, 0.06 mg; D-Biotin, 0.25 mg; folic acid, 2.5 mg; D-Pantothenic Acid, 25 mg; and nicotinamide, 50 mg. ^2^ Mineral premix provided the following per kg of diet: Fe (FeSO_4_·H_2_O), 100.0 mg; Cu (CuSO_4_·5H_2_O), 6.0 mg; Zn (ZnSO_4_·H_2_O), 80.0 mg; Mn (MnSO_4_·H_2_O), 3.0 mg; I (KI), 0.15 mg; and Se (Na_2_SeO_3_), 0.26 mg. ^3^ Nutrients levels were calculated values.

**Table 4:** Nutritional contents of FM used in experiment, % (as-fed basis).

Items	FM 1	FM 2	FM 3	FM 4	FM 5	FM 6	FM 7	FM 8	FM 9	FM10	Mean	CV	NRC
DM	92.00	92.10	92.10	90.90	93.50	93.30	93.70	92.30	92.30	91.80	92.40	0.93	93.70
CP	66.91	67.64	68.83	68.89	62.12	63.00	62.96	61.72	58.61	61.22	64.19	5.60	63.28
EE	8.40	9.10	9.00	7.80	12.00	12.40	12.20	10.00	11.00	10.80	10.27	16.14	9.71
Ash	16.70	14.90	14.90	15.30	19.30	19.50	19.10	21.20	22.50	21.10	18.45	15.27	16.07
Ca	3.63	3.00	3.02	3.30	5.04	5.26	5.16	3.16	5.45	3.08	4.01	26.62	4.28
P	2.62	2.38	2.44	2.52	3.14	3.29	3.24	2.30	3.10	2.24	2.73	15.30	2.93
Essential AAs													
Arg	3.75	3.81	3.78	3.77	3.59	3.71	3.75	3.12	3.44	3.05	3.58	7.88	3.84
His	1.85	2.41	2.45	2.04	1.40	1.38	1.41	1.39	1.49	1.35	1.72	25.62	1.44
Ile	2.90	2.78	2.72	2.91	2.41	2.36	2.39	2.56	2.33	2.51	2.59	8.66	2.65
Leu	4.87	4.84	4.82	4.93	4.13	4.09	4.07	4.36	3.90	4.27	4.43	8.94	4.47
Lys	5.21	5.23	5.18	5.29	4.48	4.47	4.48	4.36	4.15	4.27	4.71	9.68	4.56
Met	1.86	1.81	1.87	2.01	1.53	1.59	1.58	1.70	1.57	1.64	1.72	9.44	1.73
Phe	2.73	2.62	2.57	2.68	2.29	2.26	2.28	2.26	2.27	2.29	2.43	8.17	2.47
Thr	2.84	2.86	2.85	2.93	2.54	2.47	2.50	2.49	2.38	2.48	2.63	7.91	2.58
Trp	0.69	0.67	0.67	0.72	0.52	0.52	0.49	0.58	0.52	0.57	0.60	14.24	0.63
Val	3.26	3.17	3.11	3.26	2.73	2.73	2.76	2.89	2.74	2.84	2.95	7.66	3.06
Nonessential AAs													
Ala	4.24	4.28	4.22	4.27	3.91	3.97	3.95	3.74	3.96	3.70	4.02	5.38	3.93
Asp	6.08	6.04	6.04	6.16	5.50	5.46	5.46	5.40	5.17	5.32	5.66	6.57	5.41
Cys	0.58	0.65	0.66	0.67	0.53	0.56	0.53	0.53	0.54	0.68	0.59	10.84	0.61
Glu	9.23	8.90	8.88	9.28	8.59	8.53	8.56	8.49	8.12	8.33	8.69	4.32	7.88
Gly	4.15	4.14	4.12	4.06	4.80	4.97	4.89	3.55	4.74	3.46	4.29	12.64	4.71
Pro	2.88	2.76	2.72	2.72	3.37	3.26	3.31	2.11	3.21	2.39	2.87	14.57	2.89
Ser	2.43	2.59	2.62	2.51	2.32	2.32	2.31	2.15	2.19	2.12	2.36	7.54	2.43
Tyr	2.26	2.10	2.16	2.17	1.81	1.94	1.91	1.81	1.83	1.84	1.98	8.70	1.88

**Table 5:** Apparent ileal digestibility (AID) of CP and AAs in FM in experiment 1 ^1^, %.

Items	FM 1	FM 2	FM 3	FM 4	FM 5	FM 6	FM 7	FM 8	FM 9	FM 10	Mean	SEM	*p*-Value	NRC
CP	63.53 ^a^	78.09 ^a^	74.52 ^a^	76.15 ^a^	67.45 ^a^	77.86 ^a^	72.69 ^a^	48.01 ^b^	64.41 ^a^	62.19 ^ab^	68.49	3.23	<0.01	82
Essential AAs														
Arg	85.36 ^abc^	89.88 ^ab^	87.34 ^ab^	88.54 ^ab^	84.58 ^abc^	91.48 ^a^	86.57 ^ab^	77.90 ^c^	83.11 ^bc^	82.93 ^bc^	85.77	1.63	<0.01	85
His	74.61 ^ab^	86.28 ^a^	79.44 ^ab^	84.65 ^a^	73.56 ^ab^	83.74 ^a^	75.59 ^ab^	67.99 ^b^	75.90 ^ab^	72.83 ^ab^	77.46	3.10	0.03	82
Ile	80.33 ^abcd^	86.69 ^abc^	82.54 ^abc^	87.02 ^ab^	78.06 ^cd^	87.85 ^a^	83.16 ^abc^	72.25 ^d^	78.60 ^cd^	79.16 ^bcd^	81.57	1.77	<0.01	82
Leu	82.84 ^ab^	88.67 ^ab^	84.70 ^ab^	87.33 ^ab^	80.52 ^bc^	89.45 ^a^	84.89 ^ab^	73.54 ^c^	81.98 ^b^	81.48 ^b^	83.54	1.56	<0.01	82
Lys	85.06 ^abc^	90.84 ^a^	87.78 ^abc^	90.26 ^a^	84.25 ^abc^	90.88 ^a^	86.48 ^abc^	76.53 ^d^	83.11 ^bcd^	82.55 ^bcd^	85.77	1.38	<0.01	85
Met	82.92 ^cd^	90.99 ^ab^	89.10 ^abc^	89.94 ^ab^	84.88 ^bc^	92.10 ^a^	88.12 ^abc^	77.70 ^d^	85.66 ^bc^	87.72 ^abc^	86.91	1.33	<0.01	86
Phe	76.12 ^c^	85.59 ^ab^	80.95 ^abc^	84.77 ^abc^	77.47 ^bc^	87.05 ^a^	81.26 ^abc^	65.88 ^d^	78.09 ^bc^	78.34 ^abc^	79.55	1.86	<0.01	80
Thr	73.78 ^b^	82.81 ^ab^	77.15 ^ab^	82.82 ^ab^	73.47 ^b^	85.73 ^a^	78.13 ^ab^	60.93 ^c^	75.48 ^b^	74.93 ^b^	76.52	2.16	<0.01	78
Trp	71.15 ^ab^	80.11 ^ab^	75.96 ^ab^	77.55 ^ab^	67.50 ^b^	80.35 ^a^	71.92 ^ab^	53.04 ^c^	69.87 ^ab^	69.84 ^ab^	71.73	2.45	<0.01	73
Val	77.60 ^a^	84.59 ^a^	80.60 ^a^	84.24 ^a^	77.52 ^a^	85.53 ^a^	79.95 ^a^	66.40 ^b^	78.20 ^a^	78.16 ^a^	79.28	1.88	<0.01	81
Nonessential AAs														
Ala	76.79 ^ab^	84.92 ^a^	80.39 ^a^	83.92 ^a^	77.37 ^ab^	86.21 ^a^	81.71 ^a^	68.46 ^b^	77.35 ^ab^	77.87 ^ab^	79.50	2.08	<0.01	79
Asp	71.01 ^abcd^	80.54 ^abc^	76.56 ^abcd^	78.38 ^abcd^	69.08 ^bcd^	82.69 ^a^	71.90 ^abcd^	49.29 ^e^	66.31 ^d^	69.16 ^bcd^	73.96	2.67	<0.01	71
Cys	61.64 ^bcd^	65.70 ^abc^	54.87 ^cd^	78.17 ^a^	63.56 ^bc^	61.60 ^bcd^	47.77 ^d^	60.82 ^bcd^	70.40 ^ab^	63.42 ^bc^	62.80	2.87	<0.01	62
Glu	81.77 ^ab^	86.53 ^ab^	82.81 ^ab^	86.48 ^ab^	79.201 ^b^	88.96 ^a^	84.19 ^ab^	70.93 ^c^	79.89 ^b^	79.29 ^b^	82.01	1.54	<0.01	79
Gly	58.84 ^abc^	73.81 ^ab^	68.74 ^ab^	68.07 ^b^	65.87 ^abc^	78.86 ^a^	72.42 ^ab^	42.21 ^c^	62.42 ^abc^	55.97 ^bc^	64.72	4.67	<0.01	71
Pro	−24.52	19.12	14.69	6.46	−17.93	28.02	29.90	−10.09	−29.38	−26.47	−1.02	34.34	0.91	65
Ser	70.16 ^b^	79.74 ^ab^	74.70 ^ab^	80.11 ^ab^	70.55 ^b^	83.99 ^a^	75.93 ^ab^	54.65 ^c^	71.24 ^b^	69.69 ^b^	73.08	2.40	<0.01	72
Tyr	78.93 ^bcd^	85.60 ^ab^	81.09 ^abcd^	82.57 ^abc^	74.94 ^cde^	88.42 ^a^	80.28 ^abcd^	67.21 ^e^	77.71 ^bcd^	72.53 ^de^	82.09	1.99	<0.01	73

^a–e^ Within the same row, values without a common letter are significantly different (*p* < 0.05). ^1^ Data expressed as the least squares mean (n = 6) with SEM.

**Table 6:** Standardized ileal digestibility (SID) ^1^ of CP and AAs in FM in experiment 1 ^2^, %.

Items	FM 1	FM 2	FM 3	FM 4	FM 5	FM 6	FM 7	FM 8	FM 9	FM 10	Mean	SEM	*p*-Value	NRC
CP	82.61 ^abc^	95.45 ^ab^	91.67 ^ab^	94.44 ^ab^	85.59 ^ab^	97.47 ^a^	90.33 ^ab^	66.2^6 c^	82.71 ^ab^	80.41 ^bc^	86.69	3.23	<0.01	85
Essential AAs														
Arg	96.22 ^ab^	101.47 ^a^	98.81 ^a^	100.55 ^a^	96.18 a^b^	102.47 ^a^	97.55 a^b^	89.63 ^b^	95.11 ^ab^	96.33 ^ab^	97.43	1.63	<0.01	86
His	86.64 ^ab^	94.68 ^ab^	89.82 ^ab^	95.91 ^ab^	86.16 ^ab^	96.97 ^a^	89.90 ^ab^	80.90 ^b^	89.13 ^ab^	86.41 ^ab^	89.65	3.10	0.03	84
Ile	88.54 ^abc^	95.01 ^ab^	90.86 ^ab^	94.67 ^ab^	88.55 ^abc^	96.84 ^a^	92.44 ^ab^	80.70 ^c^	88.72 ^abc^	88.15 ^bc^	90.45	1.77	<0.01	83
Leu	90.41 ^ab^	96.18 ^ab^	92.09 ^ab^	95.09 ^ab^	90.03 ^ab^	97.41 ^a^	92.99 ^ab^	81.50 ^c^	90.77 ^ab^	89.65 ^b^	91.61	1.56	<0.01	83
Lys	90.20 ^abc^	95.94 ^ab^	92.96 ^abc^	95.24 ^ab^	90.05 ^abc^	96.33 ^a^	91.88 ^abc^	82.03 ^d^	89.19 ^bc^	88.20 ^cd^	91.20	1.38	<0.01	86
Met	87.49 ^cd^	96.03 ^ab^	93.84 ^abc^	93.35 ^abc^	90.03 ^abc^	95.58 ^ab^	92.77 ^ab^	82.64 ^d^	89.71 ^abc^	91.06 ^abc^	91.25	1.33	<0.01	87
Phe	85.32 ^b^	93.90 ^ab^	89.26 ^ab^	92.94 ^ab^	86.84 ^ab^	95.92 ^a^	90.15 ^ab^	74.91 ^c^	87.63 ^ab^	86.92 ^ab^	88.38	1.86	<0.01	82
Thr	86.02 ^b^	95.24 ^a^	89.77 ^ab^	94.88 ^ab^	87.16 ^b^	98.35 ^a^	91.16 ^ab^	73.55 ^c^	88.94 ^ab^	87.36 ^b^	89.24	2.16	<0.01	81
Trp	82.57 ^b^	91.54 ^ab^	87.39 ^ab^	89.73 ^ab^	81.56 ^b^	94.41 ^a^	85.98 ^ab^	65.23 ^c^	82.93 ^ab^	82.03 ^b^	84.34	2.45	<0.01	76
Val	86.66 ^b^	93.90 ^ab^	89.67 ^ab^	93.42 ^ab^	87.48 ^ab^	95.78 ^a^	90.06 ^ab^	75.83 ^c^	88.16 ^ab^	87.34 ^ab^	88.83	1.88	<0.01	83
Nonessential AAs														
Ala	88.68 ^ab^	96.93 ^a^	92.39 ^a^	95.46 ^a^	89.38 ^ab^	97.97 ^a^	93.04 ^a^	80.47 ^b^	89.60 ^ab^	89.64 ^ab^	91.36	2.09	<0.01	80
Asp	79.97 ^abc^	89.76 ^ab^	86.05 ^abc^	88.30 ^abc^	78.93 ^abc^	91.97 ^a^	81.67 ^abc^	58.44 ^d^	76.31 ^c^	78.37 ^bc^	80.98	2.67	<0.01	73
Cys	81.63 ^bcd^	86.35 ^abcd^	77.00 ^cd^	94.00 ^ab^	80.76 ^bcd^	99.59 ^a^	75.93 ^cd^	72.73 ^d^	88.49 ^abc^	83.42 ^bcd^	83.99	2.87	<0.01	64
Glu	89.50 ^ab^	94.70 ^ab^	90.99 ^ab^	94.39 ^ab^	87.96 ^b^	96.69 ^a^	91.85 ^ab^	78.59 ^c^	88.35 ^b^	87.46 ^b^	90.05	1.55	<0.01	80
Gly	89.72 ^ab^	104.68 ^a^	99.30 ^a^	100.28 ^a^	91.21 ^ab^	103.55 ^a^	95.40 ^ab^	74.08 ^b^	87.54 ^ab^	89.27 ^ab^	93.50	4.67	<0.01	75
Pro	149.27	217.73	206.45	188.79	164.40	196.54	176.24	175.27	139.13	168.66	178.25	34.34	0.89	86
Ser	85.37 ^b^	94.95 ^ab^	89.90 ^ab^	95.31 ^ab^	86.32 ^b^	98.43 ^a^	90.61 ^ab^	70.13 ^c^	87.01 ^b^	85.17 ^b^	88.32	2.40	<0.01	75
Tyr	92.34 ^abc^	99.01 ^ab^	94.21 ^abc^	96.58 ^abc^	91.60 ^abc^	101.00 ^a^	93.69 ^abc^	79.54 ^d^	90.55 ^bc^	87.57 ^cd^	92.61	1.99	<0.01	74

^a–d^ Within the same row, values without a common letter are significantly different (*p* < 0.05). ^1^ Values for the SID were calculated by correcting the values for AID for basal ileal endogenous losses. Basal ileal endogenous losses were determined (g/kg of DMI) as CP, 27.73; Arg, 1.03; His, 0.53; Ile, 0.57; Leu, 0.93; Lys, 0.63; Met, 0.24; Phe, 0.52; Thr, 0.81; Trp, 0.18; Val, 0.71; Ala, 1.20; Asp, 1.29; Cys, 0.31; Glu, 1.71; Gly, 2.96; Pro, 11.12; Ser, 0.85; and Tyr, 0.62. ^2^ Data expressed as the least squares mean (n = 6) with SEM.

**Table 7:** Prediction equations for SID Lys, Met, Thr, and Trp based on the chemical properties of 10 FM in experiment 1 ^1^, %.

Equations	Prediction Equations	R^2^	*p*-Value
1	Lys = 47.42 + 12.15 × Arg (%)	0.72	<0.01
2	Lys = 33.93 + 11.54 × Arg (%) + 26.41 × Cys (%)	0.91	<0.01
3	Lys = 29.00 + 8.31 × Arg (%) + 37.87 × Cys (%) + 3.35 × Pro (%)	0.96	<0.01
4	Met = 54.21 + 10.51 × Arg (%)	0.57	0.01
5	Thr = 25.11 + 18.01 × Arg (%)	0.53	0.02
6	Trp = 6.15 + 22.08 × Arg (%)	0.54	0.02

^1^ Equations based on analyzed nutrient content expressed on an as-fed basis, n = 10.

**Table 8:** Serum antioxidant capacity and immune function-associated indexes of piglets fed different FM in experiment 1 ^1^.

Items	FM 1	FM 2	FM 3	FM 4	FM 5	FM 6	FM 7	FM 8	FM 9	FM 10	SEM	*p*-Value
T-AOC, U/mL	1.61	2.05	2.06	2.89	2.30	1.64	2.11	1.83	2.81	2.83	0.43	0.32
GSH-Px, U/mL	451.86	510.52	437.15	381.34	426.38	419.76	394.51	440.64	396.31	380.46	31.12	0.16
MDA, nmol/mL	7.01	7.74	11.75	6.05	10.01	7.43	5.72	10.47	6.39	7.82	1.24	0.05
T-SOD, U/mL	72.28	68.69	67.16	73.04	73.17	73.02	74.74	70.45	74.36	74.68	2.75	0.64
CAT, U/mL	26.78	33.18	47.36	28.26	20.96	19.87	26.99	31.97	17.98	39.64	11.04	0.81
IgA, ug/ml	22.77	22.27	24.35	25.94	25.90	23.53	26.68	23.18	27.01	25.51	2.28	0.87
IgM, ug/ml	18.05 ^b^	30.35 ^a^	21.46 ^ab^	22.88 ^ab^	24.34 ^ab^	20.79 ^ab^	29.41 ^ab^	20.83 ^ab^	20.38 ^ab^	20.15 ^ab^	2.48	0.04
IgG, ug/ml	136.91 ^ab^	136.51 ^ab^	108.44 ^ab^	173.52 ^ab^	253.82 ^a^	170.12 ^ab^	82.93 ^b^	179.11 ^ab^	200.46 ^ab^	103.60 ^b^	27.12	0.01
IFN-γ, pg/ml	1272.73 ^ab^	924.29 ^ab^	765.69 ^b^	1499.92 ^a^	1276.21 ^ab^	1430.50 ^a^	958.06 ^ab^	1165.49 ^ab^	1351.71 ^a^	715.42 ^b^	122.62	<0.01
IL-2, pg/ml	228.58 ^bcd^	178.53 ^d^	148.51 ^d^	321.80 ^ab^	192.40 ^bcd^	281.22 ^bc^	167.42 ^d^	301.11 ^ab^	379.86 ^a^	282.95 ^bc^	19.58	<0.01
IL-6, ng/L	654.47 ^ab^	305.36 ^cd^	884.67 ^a^	662.08 ^ab^	443.80 ^bcd^	633.48 ^ab^	268.59 ^d^	628.58 ^ab^	708.14 ^ab^	566.80 ^bc^	57.08	<0.01

^a–d^ Within the same row, values without a common letter are significantly different (*p* < 0.05). ^1^ Data expressed as the least squares mean (n = 5) with SEM.

**Table 9:** Apparent total tract digestibility (ATTD) of nutrients in FM in experiment 2 ^1^, %.

Items	FM 1	FM 2	FM 3	FM 4	FM 5	FM 6	FM 7	FM 8	FM 9	FM 10	Mean	SEM	*p*-Value	NRC
DM	83.65 ^ab^	83.44 ^ab^	80.43 ^ab^	82.10 ^ab^	79.63 ^ab^	73.89 ^ab^	71.09 ^b^	75.90 ^ab^	74.94 ^ab^	84.54 ^a^	78.96	2.47	0.01	-
CP	94.65 ^a^	94.44 ^a^	91.93 ^ab^	93.62 ^ab^	93.32 ^ab^	91.22 ^ab^	89.58 ^b^	91.41 ^ab^	92.53 ^ab^	94.17 ^a^	92.69	0.83	<0.01	-
EE	68.94	82.24	69.32	69.31	81.46	74.06	71.22	75.99	70.61	83.58	74.67	4.41	0.12	-
Ash	68.21 ^ab^	67.88 ^ab^	65.20 ^ab^	69.99 ^a^	60.29 ^ab^	56.31 ^ab^	46.58 ^b^	60.03 ^ab^	55.62 ^ab^	74.03 ^a^	62.41	4.34	0.01	-
Ca	55.88	52.29	53.49	53.78	53.29	50.62	40.34	49.60	49.18	62.41	52.09	5.64	0.56	-
P	70.19	68.65	66.43	68.87	66.39	61.72	55.88	63.15	58.74	74.41	65.44	4.04	0.12	79

^a,b^ Within the same row, values without a common letter are significantly different (*p* < 0.05). ^1^ Data expressed as the least squares mean (n = 5) with SEM.

**Table 10:** N balance and deposition in FM in experiment 2 ^1^.

Items	FM 1	FM 2	FM 3	FM 4	FM 5	FM 6	FM 7	FM 8	FM 9	FM 10	SEM	*p*-Value
N intake, g/d	33.35	34.19	31.56	27.53	43.12	37.09	35.65	41.38	35.31	34.22	8.84	0.99
Fecal N, g/d	2.66	2.84	3.44	3.00	3.23	4.13	4.17	4.06	3.36	2.88	0.46	0.36
Urine N, g/d	14.11	12.22	6.47	8.49	8.03	7.39	11.60	16.35	9.14	13.53	4.37	0.89
N deposition, g/d	16.58	19.14	21.65	16.03	31.85	25.58	19.89	20.97	22.80	17.80	6.39	0.94
N deposition rate, %	50.60	53.48	66.43	55.47	71.47	67.49	52.28	52.23	59.39	54.83	8.01	0.77
BV, %	55.95	60.21	77.47	63.35	78.82	76.56	60.30	58.48	66.18	61.44	8.41	0.64

^1^ Data expressed as the least squares mean (n = 5) with SEM.

**Table 11:** Daily feces output and daily energy balance in piglets fed different FM diets in experiment 2 ^1^ (as-fed basis).

Items	FM 1	FM 2	FM 3	FM 4	FM 5	FM 6	FM 7	FM 8	FM 9	FM10	SEM	*p*-Value
Daily feces output, kg/d	0.27	0.27	0.30	0.28	0.29	0.37	0.36	0.37	0.33	0.26	0.04	0.47
Daily urine output, kg/d	2.04	2.09	3.63	2.59	2.43	2.39	2.63	3.18	3.08	3.05	0.72	0.87
Daily balance of GE												
GE intake, Mcal/d	4.39	4.36	4.40	4.53	4.60	4.70	4.49	4.63	4.50	4.38	0.67	1.00
GE in feces, Mcal/d	0.34	0.34	0.39	0.36	0.39	0.48	0.46	0.47	0.42	0.33	0.05	0.36
GE in urine, Mcal/d	0.08	0.06	0.06	0.06	0.07	0.12	0.07	0.09	0.08	0.07	0.01	0.13

^1^ Data expressed as the least squares mean (n = 5) with SEM.

**Table 12:** ATTD of GE, DE, and ME in different FM in experiment 2 ^1^ (as-fed basis).

Items	FM 1	FM 2	FM 3	FM 4	FM 5	FM 6	FM 7	FM 8	FM 9	FM 10	Mean	SEM	*p*-Value	NRC
DE, Mcal/kg	3.63 ^ab^	3.84 ^ab^	3.79 ^ab^	3.91 ^a^	3.79 ^ab^	3.58 ^ab^	3.48 ^ab^	3.62 ^ab^	3.38 ^b^	3.90 ^a^	3.69	0.11	0.01	3.96
ME, Mcal/kg	3.48 ^ab^	3.74 ^ab^	3.71 ^ab^	3.80 ^a^	3.71 ^ab^	3.44 ^ab^	3.38 ^ab^	3.50 ^ab^	3.25 ^b^	3.78 ^a^	3.58	0.12	0.02	3.53
ATTD of GE, %	81.68 ^ab^	83.40 ^ab^	82.54 ^ab^	89.08 ^a^	83.10 ^ab^	79.46 ^ab^	76.21 ^b^	83.87 ^ab^	79.40 ^ab^	89.60 ^a^	82.83	2.35	0.01	-
ME/DE, %	95.88	97.32	97.87	97.16	97.77	96.03	96.98	96.50	96.00	97.06	96.86	1.00	0.85	89.14

^a,b^ Within the same row, values without a common letter are significantly different (*p* < 0.05). ^1^ Data expressed as the least squares mean (n = 5) with SEM.

**Table 13:** Prediction equations of DE and ME in FM in experiment 2 ^1^, Mcal/kg.

Equations	Prediction Equations	R^2^	*p*-Value
Excluding AAs			
7	DE = 4.87 − 0.43 × P (%)	0.74	<0.01
8	DE = 2.51 − 0.29 × P (%) + 0.03 × CP (%)	0.90	<0.01
9	ME = 4.81 − 0.45 × P (%)	0.73	<0.01
10	ME = 2.35 − 0.30 × P (%) + 0.03 × CP (%)	0.88	<0.01
11	ME = 1.75 − 0.39 × P (%) + 0.04 × CP (%) + 0.04 × EE (%)	0.95	<0.01
Including AAs			
12	DE = 1.86 + 3.02 × Cys (%)	0.86	<0.01
13	ME = 1.66 + 3.17 × Cys (%)	0.84	<0.01

^1^ Equations based on analyzed nutrient content expressed on an as-fed basis (n = 10).

## Data Availability

Due to ethical restrictions, the raw data cannot be made publicly available. However, de-identified data may be obtained from the corresponding author upon reasonable request.

## Abbreviations

The following abbreviations are used in this manuscript:

AA	Amino acid
AID	Apparent ileal digestibility
ATTD	Apparent total tract digestibility
BV	Biological value
Ca	Calcium
CV	Coefficient of variation
CP	Crude protein
DE	Digestible energy
DM	Dry matter
EAA	Essential amino acid
EE	Ether extract
FM	Fishmeal
GE	Gross energy
LAA	Limiting amino acid
ME	Metabolizable energy
N	Nitrogen
P	Phosphorus
SID	Standardized ileal digestibility

## References

[1] Garavito-Duarte Y.R., Levesque C.L., Herrick K., Perez-Palencia J.Y. (2023). Nutritional value of high protein ingredients fed to growing pigs in comparison to commonly used protein sources in swine diets. J. Anim. Sci..

[2] Jeyasanta K.I., Patterson J. (2020). Study on the effect of freshness of raw materials on the final quality of fish meals. Indian J. Mar. Sci..

[3] Kaewtapee C., Mosenthin R., Nenning S., Wiltafsky M., Schäffler M., Eklund M., Rosenfelder-Kuon P. (2018). Standardized ileal digestibility of amino acids in European soya bean and rapeseed products fed to growing pigs. J. Anim. Physiol. Anim. Nutr..

[4] Kong C., Kim K.H., Ji S.Y., Kim B.G. (2021). Energy concentration and phosphorus digestibility in meat meal, fish meal, and soybean meal fed to pigs. Anim. Biosci..

[5] Park C.S., Adeola O. (2022). Digestibility of amino acids in fish meal and blood-derived protein sources fed to pigs. Anim. Biosci..

[6] Jones A.M., Wu F., Woodworth J.C., Tokach M.D., Goodband R.D., DeRouchey J.M., Dritz S.S. (2018). Evaluating the effects of fish meal source and level on growth performance of nursery pigs. Transl. Anim. Sci..

[7] NRC (2012). Nutrient Requirements of Swine.

[8] Stein H.H., Shipley C.F., A Easter R. (1998). Technical note: A technique for inserting a T-cannula into the distal ileum of pregnant sows. J. Anim. Sci..

[9] Park C.S., Adeola O. (2020). Basal ileal endogenous losses of amino acids in pigs determined by feeding nitrogen-free diet or low-casein diet or by regression analysis. Anim. Feed. Sci. Tech..

[10] Li S., Sauer W.C., Fan M.Z. (1993). The effect of dietary crude protein level on amino acid digestibility in early-weaned piglets. J. Anim. Physiol. Anim. Nutr..

[11] Yu J., Yu G., Yu B., Zhang Y., He J., Zheng P., Mao X., Luo J., Huang Z., Luo Y. (2020). Dietary protease improves growth performance and nutrient digestibility in weaned piglets fed diets with different levels of soybean meal. Livest. Sci..

[12] AOAC International (2019). Official Methods of Analysis of AOAC International.

[13] Stein H., Fuller M., Moughan P., Sève B., Mosenthin R., Jansman A., Fernández J., de Lange C. (2007). Definition of apparent, true, and standardized ileal digestibility of amino acids in pigs. Livest. Sci..

[14] Adeola O. (2001). Digestion and balance techniques in pigs. Swine Nutrition.

[15] Goethals, Rijpert J.H.M., Spek J.W., Millet S., Bikker P. (2023). Amino acid requirement of weaned piglets. Wageningen UR Livestock Research Rapport 1436.2023.

[16] Jansman A.J.M., Cirot O., Corrent E., Lambert W., Ensink J., van Diepen J.T.M. (2019). Interaction and imbalance between indispensable amino acids in young piglets. Animal.

[17] Rojas O.J., Stein H.H. (2013). Concentration of digestible, metabolizable, and net energy and digestibility of energy and nutrients in fermented soybean meal, conventional soybean meal, and fish meal fed to weanling pigs. J. Anim. Sci..

[18] Cervantes-Pahm S.K., Stein H.H. (2010). Ileal digestibility of amino acids in conventional, fermented, and enzyme-treated soybean meal and in soy protein isolate, fish meal, and casein fed to weanling pigs. J. Anim. Sci..

[19] Moughan P.J., Leenaars G.S.M. (1992). Endogenous amino acid flow in the stomach and small intestine of the young growing pig. J. Sci. Food Agric..

[20] Livingstone R., McWilliam R. (1985). The effect of terminal ileum cannulation on the performance of growing pigs. Br. Vet. J..

[21] Zebrowska T., Buraczewski S. (1998). Methods for determination of amino acids bioavailability of pigs-review. Asian-Australas. J. Anim. Sci..

[22] Bacou E., Walk C., Rider S., Litta G., Perez-Calvo E. (2021). Dietary oxidative distress: A review of nutritional challenges as models for poultry, swine and fish. Antioxidants.

[23] Ighodaro O.M., Akinloye O.A. (2018). First line defence antioxidants-superoxide dismutase (SOD), catalase (CAT) and glutathione peroxidase (GPX): Their fundamental role in the entire antioxidant defence grid. Alex. J. Med..

[24] Hao Y., Xing M., Gu X. (2021). Research progress on oxidative stress and its nutritional regulation strategies in pigs. Animals.

[25] Grönwall C., Vas J., Silverman G.J. (2012). Protective roles of natural IgM antibodies. Front Immunol..

[26] Eriguchi Y., Nakamura K., Yokoi Y., Sugimoto R., Takahashi S., Hashimoto D., Teshima T., Ayabe T., Selsted M.E., Ouellette A.J. (2018). Essential role of IFN-γ in T cell–associated intestinal inflammation. JCI Insight.

[27] Fritsch S.D., Weichhart T. (2016). Effects of interferons and viruses on metabolism. Front. Immunol..

[28] Hoyer K.K., Dooms H., Barron L., Abbas A.K. (2008). Interleukin-2 in the development and control of inflammatory disease. Immunol. Rev..

[29] Wu G., Bazer F.W., Davis T.A., Kim S.W., Li P., Rhoads J.M., Satterfield M.C., Smith S.B., Spencer T.E., Yin Y. (2009). Arginine metabolism and nutrition in growth, health and disease. Amino Acids.

[30] Kim B.G., Liu Y., Stein H.H. (2014). Energy concentration and phosphorus digestibility in yeast products produced from the ethanol industry, and in brewers’ yeast, fish meal, and soybean meal fed to growing pigs. J. Anim. Sci..

[31] Kim S.W., A Easter R. (2001). Nutritional value of fish meals in the diet for young pigs. J. Anim. Sci..

[32] Sauvant D., Perez J.M., Tran G. (2004). Tables of Composition and Nutritional Value of Feed Materials: Pigs, Poultry, Cattle, Sheep, Goats, Rabbits, Horses and Fish.

[33] Ouyang Y.N., Wang S.Y., Liang J.C., Xue B., Li W.J., Li Y.J., Hong Q.H. (2021). Effect of dietary phosphorus levels on energy metabolism and serum reproductive hormones of Yunnan semi-fine wool sheep during non-pregnancy period. Feed. Res..

[34] Zong E., Huang P., Zhang W., Li J., Li Y., Ding X., Xiong X., Yin Y., Yang H. (2018). The effects of dietary sulfur amino acids on growth performance, intestinal morphology, enzyme activity, and nutrient transporters in weaning piglets. J. Anim. Sci..

